# Blood Oxygenation Level-Dependent MRI to Assess Renal Oxygenation in Renal Diseases: Progresses and Challenges

**DOI:** 10.3389/fphys.2016.00667

**Published:** 2017-01-05

**Authors:** Menno Pruijm, Bastien Milani, Michel Burnier

**Affiliations:** Service of Nephrology and Hypertension, Department of Medicine, Centre Hospitalier Universitaire VaudoisLausanne, Switzerland

**Keywords:** BOLD-MRI, chronic kidney disease, renal artery stenosis, furosemide, TLCO-technique

## Abstract

BOLD-MRI (blood oxygenation-level dependent magnetic resonance imaging) allows non-invasive measurement of renal tissue oxygenation in humans, without the need for contrast products. BOLD-MRI uses the fact that magnetic properties of hemoglobin depend of its oxygenated state:: the higher local deoxyhemoglobin, the higher the so called apparent relaxation rate R2^*^ (sec^−1^), and the lower local tissue oxygen content. Several factors other than deoxyhemoglobin (such as hydration status, dietary sodium intake, and susceptibility effects) influence the BOLD signal, and need to be taken into account when interpreting results. The last 5 years have witnessed important improvements in the standardization of these factors, and the appearance of new, highly reproducible analysis techniques of BOLD-images, that are reviewed in this article. Using these new BOLD-MRI analysis techniques, it has recently been shown that persons suffering from chronic kidney diseases (CKD) have lower cortical oxygenation than normotensive controls, thus confirming the chronic hypoxia hypothesis. The acute alterations in R2^*^ after the administration of furosemide are smaller in CKD, and represent an estimate of the oxygen-dependent tubular transport of sodium. BOLD-MRI-alone or in combination with other functional MRI methods- can be used to monitor the renal effects of drugs, and is increasingly used in the preclinical setting. The near future will tell whether or not BOLD-MRI represents a new tool to predict renal function decline an adverse renal outcome.

## Introduction

Chronic kidney disease (CKD), defined as an estimated glomerular filtration rate below 60 ml/min/1.73 m^2^ and/or the presence of (micro) albuminuria, has become a major public health problem with a global prevalence in the general population of ~10% (Ponte et al., 2013). CKD is an independent cardiovascular risk factor and associated with increased mortality (Astor et al., 2011). The pathophysiology of CKD and its progression to end stage renal disease is complex and incompletely understood, but mounting evidence from animal studies suggests that renal tissue hypoxia is the final and common pathway, irrespective of etiology (Alberti and Zimmet, 1998; Fine and Norman, 2008). According to this “chronic hypoxia hypothesis,” loss of peritubular capillaries induces interstitial hypoxia which triggers local inflammation and fibrosis. This leads in turn to further obliteration and loss of capillaries, thus completing the vicious circle. So far, evidence for the chronic hypoxia hypothesis in humans has been sparse, mainly because of the lack of methods to assess renal tissue oxygenation in a reliable, non-invasive manner.

A technique to measure tissue oxygenation in humans would be a valuable tool for several reasons. First of all, such a technique could be used to confirm or reject the chronic hypoxia hypothesis. Secondly, ideally it would allow to identify CKD patients at increased risk for rapid renal function decline and end-stage renal disease, since according to the chronic hypoxia hypothesis, those with the lowest degree of oxygenation have the highest renal risk. Finally, drugs that chronically increase renal tissue oxygenation would have the potential to retard the progression of CKD. Thus, a method capable of measuring renal tissue oxygenation could identify at an early stage drugs with oxygen-increasing and possibly nephroprotective potential.

Since its first description in 1996 (Prasad et al., 1996), renal blood oxygenation-level dependent MRI (BOLD-MRI) is seen by many as the most promising method to assess renal oxygenation non-invasively in humans. In brief, BOLD-MRI uses the paramagnetic properties of deoxyhemoglobin to assess tissue oxygenation: the higher local deoxyhemoglobin, the higher the apparent relaxation rate R2^*^ (sec^−1^), and the lower local tissue oxygen content, assuming that blood pO_2_ is in equilibrium with tissue pO_2_. BOLD-MRI does not require the administration of (possibly nephrotoxic) contrast media, making it an interesting tool for CKD patients. BOLD-MRI is fast and can be repeated many times in short time intervals without side-effects.

Despite these advantages, BOLD-MRI is for the moment mainly used in research settings and not yet fully integrated in clinical practice. The initial enthusiasm was tempered by studies who failed to demonstrate with BOLD-MRI that chronic hypoxia is indeed present in humans. Besides, we have learned that factors other than oxygenation (such as blood pH, hematocrite, hydration status, and susceptibility effects, see below) influence the BOLD-signal (Prasad and Epstein, 1999; Pruijm et al., 2010; Neugarten, 2012). However, progress has been made in the standardization of these factors, as well as in data acquisition and analysis, which has recently lead to interesting results and will possibly lead to new applications for this technique. In this article, we review the technical hurdles that had to be overcome, others that still need to be resolved, the main results of clinical studies and the perspectives of BOLD-MRI.

## BOLD-MRI: technique and pitfalls

The basic principle of Magnetic Resonance Imaging (MRI) can be summarized as follows: when atomic nuclei with non-zero angular momentum (like hydrogen) are placed in a magnetic field and reach thermal equilibrium, an excess of nuclei lying in the low energy state appears. This excess of nuclei can be brought to some higher energy state when excited by an electromagnetic wave (pulse). The nuclei return to equilibrium after the excitation while emitting back a radiation; this takes a certain time, measured as the so called relaxation times (T1 and T2). These parameters provide information about the density and localization of (hydrogen or other) nuclei, and allow the construction of an image.

BOLD-MRI measures the apparent relaxation rate R2^*^ (or decay rate, defined as 1/T2^*^ and expressed in sec^−1^) for each voxel located in the kidney. This parameter is influenced by any kind of inhomogeneity in the static magnetic field, in particular by the effect of deoxyhemoglobin, which has a strong positive magnetic susceptibility due to its central iron atom. The susceptibility difference between deoxyhemoglobin and surrounding tissues will generate intra-voxel magnetic field inhomogeneities. As such, the decay rate R2^*^ will be enhanced when the local deoxyhemoglobin concentration is increased. Assuming that blood pO2 is in equilibrium with tissue pO2, R2^*^ values allows to qualitatively compare tissue oxygenation between different voxel of the same subject and between subjects: low R2^*^ values indicates high tissue oxygenation, and vice versa (Prasad, 2006). BOLD-MRI has been validated in animal studies, showing that R2^*^ correlates negatively with directly measured pO2 (Pedersen et al., 2005). However, several points merit attention in the interpretation of R2^*^ values. First, factors that affect the oxygen dissociation curve (which describes the relationship between de(oxy)Hb and pO2) such as blood pH, body temperature, and hematocrite alter the equilibrium described above, and should be taken into account when performing BOLD-MRI (Neugarten, 2012). Second, the BOLD-signal is sensitive to an acute water load (Prasad and Epstein, 1999). This sensitivity is partly explained by the water-induced reduction in oxygen-consuming tubular transport, but an increase in tubular volume after water intake will also reduce the local deoxyhemoglobin volume fraction and thus further decrease R2^*^. Standardization of water intake is therefore warranted. Because tubular sodium reabsorption is a major driver of oxygen consumption, short-term alterations in dietary sodium intake influence significantly medullary R2^*^ values, and urinary sodium excretion (as a proxy of sodium intake) should therefore be measured whenever possible (Pruijm et al., 2010).

**Figure 1:**
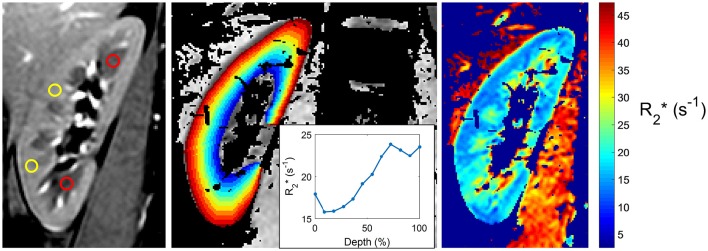
**Three currently used techniques to analyze BOLD-MRI. (Left)** Classical ROI-based technique, with placement of circle-shaped regions of interest (ROIs) in the renal cortex (yellow) and medulla (red); **(Middle)** Twelve-Layer Concentric Objects (TLCO) technique, which divides the renal parenchyma in 12 layers of equal thickness; **(Right)** Fractional hypoxia technique, which counts the percentage of voxels with an R2^*^ value >30 sec^−1^.

While the acquisition of BOLD-MRI is performed in many centers worldwide, no general consensus exists on how to analyze the BOLD-images, and many methods are in use (see Figure 1 for a few examples). In the oldest and most frequently used method, the ROI technique, small circles each containing a collection of voxels -called regions of interest (ROI's)- are placed manually in the cortex and in the medulla of each slice (Figure 1, left image). This allows the calculation of the average cortical and medullary R2^*^ values, per kidney or for both kidneys together (Simon-Zoula et al., 2006). Placement of ROIs is easy in kidneys with preserved renal function, but difficult in patients with advanced CKD due to the lack of cortico-medullary differentiation in the latter (e.g., the human eye is no longer capable of differentiating the cortex from the medulla purely based on radiological contrast differences). A second, recent method called the TLCO (12 layers concentric objects) technique is a semi-automatic procedure that divides the kidney in 12 layers of equal thickness. The mean R2^*^ values of all layers can be plotted as a curve (the R2^*^ radial profile) with a certain slope (Figure 1, middle image). The steepness of the slope is associated with the degree of CKD: the higher the eGFR, the steeper the slope (Milani et al., 2016). The R2^*^ profile has a low inter-observer variability and can visualize layer by layer the effect of external stimuli, making it an interesting tool to study the effects of drugs, as recently demonstrated (Vakilzadeh et al., 2015).

Since the BOLD-signal is influenced by many factors, of which some are scanner related, absolute R2^*^ values are not comparable across sites. The steepness of the R2^*^ slopes less of the R2^*^ values, but merely of their intra-compartmental distribution. This observable may therefore be more suitable for comparisons across centers, which would be a huge advantage for eventual future integration in clinical practice. Another way to overcome this hurdle is to perform a dynamic test (such as the administration of IV furosemide or 100% oxygen breathing), and to assess the percentage change in R2^*^.

In a third method called the fractional tissue hypoxia-technique, the whole renal parenchyma is selected, and the percentage of R2^*^ values above a certain threshold (usually 30 s^−1^ or >2.5 standard deviations of the average R2^*^ value) is reported (Saad et al., 2013). This technique provides a variable—the percentage of hypoxic tissue- that can be easily interpreted by clinicians, but it does not differentiate between cortex and medulla (Figure 1, right image).

Taken together, each technique has his advantages and disadvantages. The most promising and reproducible technique for CKD patients is at the moment, to our opinion, the TLCO method; international efforts are ongoing and necessary to standardize analysis methods in the years to come.

## BOLD-MRI in CKD

Surprisingly, BOLD-MRI studies have not uniformly demonstrated that renal tissue oxygenation is reduced in CKD patients as compared with controls. Some studies reported higher R2^*^ values suggesting lower renal oxygenation at lower eGFR, whereas other studies did not find any correlation between CKD status and R2^*^ (Table 1). Unfortunately, many of the “early” studies lacked information on drug-or sodium intake, did not standardize water intake, or did not differentiate between the underlying causes of CKD. However, even well-designed studies failed to show differences in R2^*^ between persons with and without CKD (Khatir et al., 2015). It has become clear that the analysis technique has a large impact on the results. Our research group has recently demonstrated that the ROI technique is highly observer-dependent in advanced CKD, and should therefore not be used in these patients (Piskunowicz et al., 2015). The importance of the used analysis technique is further illustrated by the fact that our research group did not find any differences in R2^*^ with the ROI technique, whereas the use of the TLCO technique in the same patient cohort showed significant differences in R2^*^ between the cortical layers of CKD patients and controls (Milani et al., 2016).

**Table 1:** **Overview of the studies that have used BOLD-MRI to assess renal tissue oxygenation in CKD patients as compared with controls**.

**Author**		***N***	**Design**	**Field strength (Tesla)**	**Analysis method**	R2^*^ cortex	R2^*^ medulla	**Remark**
Inoue	2011	119	CKD-control	1.5	ROI	No difference	Not available	Only DM
Wang	2011	27	CKD-control	1.5	ROI	No difference	Lower in CKD	Only DM
Michaely	2012	280	Observational	1.5 and 3	ROI	No difference	No difference	
Xin-Long	2012	26	CKD-control	3	ROI	Higher in CKD	Higher in CKD	
Yin	2012	115	CKD-control	3	ROI	Higher in CKD	Higher in CKD	Only DM
Pruijm	2014	195	CKD-control	3	ROI	No difference	No difference	
Vink	2015	75	Hypertensives	1.5 and 3	Fractional and Compartmental	No difference	Higher at lower eGFR	eGFR 75 ± 18 ml/min
Thacker	2015	47	CKD-control	3	Large ROI-entire parenchyma	Higher in CKD	No difference	
Prasad	2015	59	CKD-control	3	ROI	Higher in CKD	No difference	
Khatir	2015	86	CKD-control	1.5	ROI	No difference	No difference	
Milani	2016	207	CKD-control	3	TLCO	Higher in CKD	Lower in CKD	

As can be appreciated in Table 1, recent BOLD-MRI studies have actually demonstrated that CKD patients have higher R2^*^ values (corresponding to lower renal tissue oxygenation) as compared to controls, thus confirming the findings of animal studies. Differences in R2^*^ were mainly confined to the cortex, which is somewhat surprising, since the cortex has higher pO2 values than the medulla (50 vs. 10–20 mmHg), receives 90% of the renal blood flow, and has relatively lower oxygen consumption than the medulla (Aukland and Krog, 1960; Heyman et al., 2008). In theory, the cortex should therefore be better protected against hypoxia. Whether this is explained by glomerular hyperfiltration in residual glomeruli, altered diffusive shunting pathways or reduced metabolic efficiency of solute transport in tubular cells is actually unclear. Of note, although the mean R2^*^ is higher in CKD patients, differences are small, and according to some authors mainly driven by a minority of CKD patients with high R2^*^ values (Prasad et al., 2015). This finding illustrates that renal tissue oxygenation is rather tightly controlled in the majority of individuals. In this respect, some have stated that interstitial fibrosis- the consequence of hypoxia and one of the hallmarks of advanced kidney disease- might be a way to maintain to a certain degree renal oxygenation, by decreasing local oxygen-consuming active transport. Long-term 5/6 nephrectomy models in the rat have indeed reported maintained pO2 levels in remnant kidneys (Priyadarshi et al., 2002). In humans, this hypothesis has not been confirmed so far; one study reported no correlation between R2^*^ values and the degree of fibrosis (Ries et al., 2003), another found higher R2^*^ values at increasing degrees of biopsy-proven fibrosis (Inoue et al., 2011). Finally, it is actually unknown whether CKD patients with high R2^*^ values have more progressive disease than those with lower R2^*^ values, and longitudinal follow-up studies are therefore eagerly awaited.

## BOLD-MRI and drugs

As stated in the introduction, BOLD-MRI can be repeated many times without side effects, making it an interesting tool to study the effect of drugs. On this behalf, the loop diuretic furosemide has been the most investigated. Blocking the Na^+^-K^+^-2Cl^−^ transporter in the thick ascending loop of Henle with an IV bolus of furosemide leads to an acute decrease in active oxygen-consuming sodium transport, and increases local pO2 (Liss et al., 1999). As a proof of concept, acute furosemide-induced decreases in medullary R2^*^ have been reported in both animals and humans (Brezis et al., 1994; Epstein and Prasad, 2000). Of interest, the medulla of CKD patients shows smaller decreases in R2^*^ in response to furosemide than controls, and the furosemide-induced R2^*^ change correlates well with the eGFR (Pruijm et al., 2014). This is explained by some as purely a dose effect due to the reduced renal function. Indeed, in order to exert its natriuretic action, furosemide has to be secreted by the proximal tubular cells, which is less the case at lower eGFR. Others see the R2^*^ response as an indicator of tubular function and transport. For example, hypertensive individuals show, as CKD patients, a blunted R2^*^ response to furosemide, although their eGFR is preserved, an observation that possibly refers to altered tubular sodium handling in hypertensives. Whether the R2^*^ response to furosemide is indeed a marker of tubular function and mass, and has prognostic potential to predict adverse renal outcome, is currently unknown.

Few studies have tested other drugs with regard to their effect on R2^*^. One of the earliest studies reported acute increases in medullary and cortical R2^*^ after iodinated contrast products, which is in line with their well-known vasoconstrictive and nephrotoxic potential (Hofmann et al., 2006). Inversely, acute decreases in R2^*^ (within 1 h) have been described after the administration of blockers of the renin-angiotensin system in small studies that included hypertensive or CKD patients (Djamali et al., 2007; Siddiqi et al., 2014). This is in line with the nephroprotective properties of these drugs, that lower intraglomerular pressure, tubular sodium transport, local inflammation, and oxidative stress (Tocci and Volpe, 2011). In contrast, chronic intake of renin-angiotensin system inhibitors did not alter renal R2^*^ values (Pruijm et al., 2013), although a small decrease in cortical R2^*^ values was found in hypertensive patients after 2 months of aliskiren (Vakilzadeh et al., 2015). These studies show that acute alterations in R2^*^ occur, but they also illustrate once more that chronic renal tissue oxygenation is rather tightly controlled. The acute, drug induced- alterations in R2^*^ offer insights in the mechanism of action and possibly nephroprotective potential of drugs in a preclinical setting. BOLD-MRI can be combined with other MRI methods such as arterial spin labeling, capable of measuring localrenal blood flow, or dynamic contrast enhanced-imaging that can directly measure glomerular filtration rate (Ebrahimi et al., 2014). Therefore, functional MRI can provide a wealth of information in the renal mechanisms of action of drugs, and possibly indicate renal side effects or benefits long before clinical outcome studies. Considering animal studies, functional MRI also has the potential to reduce animal usage. For these reasons, we expect functional MRI to play an increasingly important role in drug research.

## BOLD-MRI and renal artery stenosis

As any organ, renal tissue oxygenation depends not only of local oxygen consumption (mainly active tubular sodium-dependent transport), but also of oxygen delivery (renal blood flow and hemoglobin level). Renal artery stenosis (RAS) is the classic example of ischemic nephropathy, and one would expect to find cortical and medullary hypoxia in this patient group. Animal studies have indeed reported acute increases in R2^*^ after clipping of the renal artery (Juillard et al., 2004). However, no hypoxia could be detected with BOLD-MRI 4 weeks after clipping of the renal artery (Rognant et al., 2010). As in the remnant kidney model, the kidneys at the side of the clipped renal artery were atrophic and non-functional, and this study therefore does not necessarily reflect the situation as often encountered in the clinic when RAS kidneys are (slightly) reduced in size, but not atrophic.

In humans, BOLD-MRI has, to the best of our knowledge, not been used in situations of acute renal artery occlusion. In chronic RAS, increases in R2^*^ have been reported in patients with severe RAS (>90%), but not in those with less severe RAS (Gloviczki et al., 2011a). Hence, renal tissue oxygenation seems to be relatively independent from global renal blood flow. Of note, kidneys have the particularity that increases in RBF do not necessarily increase local pO2. Indeed, an increase in RBF leads, at constant filtration fraction, also to a higher GFR which on its term will increase the amount of filtered sodium, and thus the tubular sodium load and oxygen consumption (Hansell et al., 2013).

Some investigators have used the R2^*^ response to furosemide as a marker of viability of renal tissue (Gloviczki et al., 2011b). In unilateral RAS, the eGFR does not provide information on the relative function of each kidney, whereas BOLD-MRI allows per-kidney analysis. The research group from the Mayo Clinic has demonstrated that the change in R2^*^ after IV furosemide is smaller in those with severe RAS and reduced renal volume than in those with moderate RAS, and that non-viable renal tissue is therefore possibly defined as tissue that does not exhibit any change in R2^*^ after furosemide (Gloviczki et al., 2011a).

## Clinical applications and perspectives

### Renal artery stenosis

Large randomized trials such as the Angioplasty and Stenting for Renal Artery Lesions trial (ASTRAL) have tempered the enthusiasm of clinicians to perform renal artery angioplasty ± stenting in RAS patients (Investigators et al., 2009). Nevertheless, it is well known that some patients definitely benefit from angioplasty, and the main question remains how to predict the outcome of this procedure. The studies outlined above show that renal oxygenation can be maintained over a wide range of stenosis, but that above a certain individual threshold, chronic ischemia occurs, leading to inflammation, and decline of renal function, and diminished response to furosemide (Gloviczki et al., 2011a,b). This suggests that patients who will most likely benefit from angioplasty present chronic ischemia and a maintained response to furosemide, but this has not been tested in clinical practice. Hermann et al. recently reported that fractional hypoxia was higher in the stenotic kidneys of patients with high-grade RAS than in patients with essential hypertension (22.1 ± 20 vs. 9.6 ± 7%). Fractional hypoxia diminished (from 22.1 ± 20 to 14.9 ± 18.3%) after renal stenting (Herrmann et al., 2016). In a similar way, an English research group showed that those with higher overall R2^*^ levels and preserved renal volume and function had favorable outcomes after revascularization (Chrysochou et al., 2012). The integration of functional BOLD-MRI in the workup and decision process of RAS opens new perspectives, but this needs further validation in clinical studies.

### Chronic kidney disease

Ideally, BOLD-MRI should identify patients at increased risk of CKD progression, expecting that those with the lowest renal oxygenation, or the lowest R2^*^ change after furosemide, have the highest risk of progression. So far, these data are lacking, hampering the introduction of BOLD-MRI in clinical practice.

Another issue is the fact that BOLD-MRI alone cannot establish whether a high R2^*^ value is the result of low oxygen delivery (low RBF, extended renal fibrosis), high oxygen consumption (glomerular hyperfiltration, enhanced tubular absorption), or both. CKD is characterized by increased fibrosis, which on the one hand limits oxygen diffusion out of capillaries into renal cells, yet on the other hand also limits oxygen consumption. Quantifying fibrosis and regional blood flow, in combination with BOLD-MRI, could therefore provide useful information. Apart from T_1_mapping, so-called diffusion-weighted MRI (DW-MRI) offers a non-invasive way to quantify renal fibrosis and microcirculation. DW-MRI collects images with and without diffusion weighted gradients, and expresses molecular diffusion as the apparent diffusion coefficient (ADC) Liss et al., 2013. The total ADC can be separated in a perfusion fraction (F_p_) and perfusion-free diffusion (ADC_D_), as measures of local microcirculation and fibrosis. A reduction in ADC has been shown to correlate with CKD staging and the degree of fibrosis (Xu et al., 2010; Zhao et al., 2014), and a reduction in F_p_ has been demonstrated in renal allografts suffering from acute rejection (Eisenberger et al., 2010). Whether a combination of BOLD-MRI and diffusion MRI is capable of predicting renal function decline is not yet known, and subject of active research.

## Conclusion

BOLD-MRI is an exciting technique to assess renal oxygenation non-invasively in humans. Increased knowledge of factors that influence the BOLD-signal has lead to better standardization, and refinements in the analysis technique to highly reproducible results. BOLD-MRI is a powerful tool to detect the influence of altered hemodynamics, drugs, or dietary factors on renal oxygenation. The near future will tell if BOLD-MRI (alone or in combination with other MRI modalities) allows the selection of RAS patients who will benefit from revascularization, and/or early identification of CKD patients at high risk for renal function decline.

## Author contributions

MP: Drafting the article, final approval of the version to be published, agrees to be accountable for all aspects of the work in ensuring that questions related to the accuracy or integrity of any part of the work are appropriately investigated and resolved. BM and MB: Revising the article critically for important intellectual content, final approval of the submitted version, both agree to be accountable for all aspects of the work in ensuring that questions related to the accuracy or integrity of any part of the work are appropriately investigated and resolved.

## Funding

This work was supported by grants from the Swiss National Science Fondation (FN 32003B-149309 and 320030-169191).

### Conflict of interest statement

The authors declare that the research was conducted in the absence of any commercial or financial relationships that could be construed as a potential conflict of interest.
